# Preimplantation expression of the somatic form of Dnmt1 suggests a role in the inheritance of genomic imprints

**DOI:** 10.1186/1471-213X-8-9

**Published:** 2008-01-25

**Authors:** M Cecilia Cirio, Sarayu Ratnam, Feng Ding, Bonnie Reinhart, Chris Navara, J Richard Chaillet

**Affiliations:** 1 Department of Molecular Genetics and Biochemistry, University of Pittsburgh School of Medicine, Pittsburgh, PA, USA; 2 Pittsburgh Development Center, Magee-Women's Research Institute, Pittsburgh, PA, USA; 3 Departments of Cell Biology and Physiology and Obstetrics-Gynecology-Reproductive Sciences, University of Pittsburgh School of Medicine, Pittsburgh, PA, USA

## Abstract

**Background:**

Identical DNA methylation differences between maternal and paternal alleles in gametes and adults suggest that the inheritance of genomic imprints is strictly due to the embryonic maintenance of DNA methylation. Such maintenance would occur in association with every cycle of DNA replication, including those of preimplantation embryos.

**Results:**

The expression of the somatic form of the Dnmt1 cytosine methyltransferase (Dnmt1s) was examined in cleavage-stage preimplantation mouse embryos. Low concentrations of Dnmt1s are found in 1-, 2-, 4-, and 8-cell embryos, as well as in morulae and blastocysts. Dnmt1s is present in the cytoplasm at all stages, and in the nuclei of all stages except the 1-cell, pronuclear-stage embryo. The related oocyte-derived Dnmt1o protein is also present in nuclei of 8-cell embryos, along with embryo-synthesized Dnmt1s. Dnmt1s protein expressed in 1-cell and 2-cell embryos is derived from the oocyte, whereas the embryo synthesizes its own Dnmt1s from the 2-cell stage onward.

**Conclusion:**

These observations suggest that Dnmt1s provides maintenance methyltransferase activity for the inheritance of methylation imprints in the early mouse embryo. Moreover, the ability of Dnmt1o and Dnmt1s proteins synthesized at the same time to substitute for one another's maintenance function, but the lack of functional interchange between oocyte- and embryo-synthesized Dnmt1 proteins, suggests that the developmental source is the critical determinant of Dnmt1 function during preimplantation development.

## Background

Genomic imprinting is an epigenetic process that distinguishes parental alleles in nuclei of diploid cells. Such an imprinted state must come about by first epigenetically distinguishing the two alleles in the parental gametes. Afterwards, oocyte- and sperm-specific epigenetic marks are perpetuated as cells divide during embryogenesis. For many reasons, DNA cytosine methylation is an excellent candidate for this epigenetic distinction [1]. First, alleles of most imprinted genes are differentially methylated in embryonic and adult tissues. Second, this DNA methylation difference first appears between alleles in the parental germ lines, suggesting that the embryo inherits patterns of DNA methylation established in the gametes. Third, the process whereby parental imprints are established de novo in the germ cells is catalyzed by a cytosine methyltransferase [2]. Lastly, the perpetuation of DNA methylation patterns on imprinted genes in the post-implantation embryo is absolutely dependent on the maintenance methyltransferase activity of the somatic variant of a different cytosine methyltransferase, Dnmt1 [3].

Although DNA methylation's function in establishing imprints in the gametes, and in perpetuating these after implantation of the embryo is well established, the role of DNA methylation during the intervening preimplantation stages of embryo development is much less certain. The Dnmt1o variant of Dnmt1, which is synthesized in oocytes, traffics from the cytoplasm to the nuclei of 8-cell embryos, where it maintains genomic imprints [4,5]. However, this maternally derived protein is excluded from the nuclear compartment of preimplantation embryos at non-8-cell stages [5], and the longer somatic variant of Dnmt1, Dnmt1s, was not detected in wild-type preimplantation embryos [6]. These observations suggest that Dnmt1s may be present in preimplantation embryos, but in concentrations that we did not detect using particular immunodetection methods [6]. An alternative possibility is that the maintenance of genomic imprints at non-8-cell stages of preimplantation development occurs by non-Dnmt1 maintenance mechanisms.

We had previously detected the Dnmt1s protein in nuclei of 4-cell, 8-cell and 16-cell embryos that developed in the absence of maternal Dnmt1o protein [7]. These observations suggested that the loss of Dnmt1o from early embryos resulted in a compensatory increase in expression of Dnmt1s protein that then trafficked readily into nuclei of many cleavage-stage embryos, where this concentrated nuclear Dnmt1 was detected by immunostaining. Because of this detection of Dnmt1s protein in embryos lacking Dnmt1o, and because of the absence of experimental support for an alternative mechanism to explain the inheritance of methylation patterns associated with imprinted alleles, we used an improved immunodetection method to explore the possibility that Dnmt1s is expressed in wild-type preimplantation embryos.

## Results

### Dnmt1s is present in fully grown oocytes

**Figure 1:**
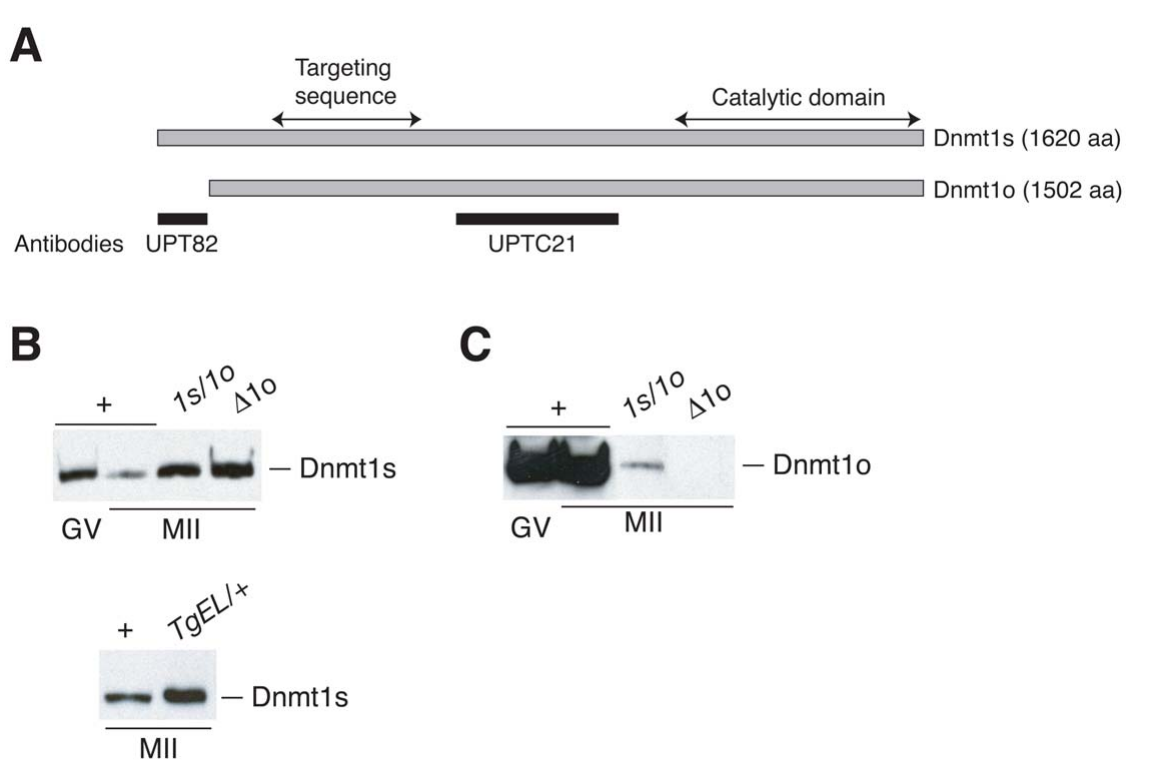
**Dnmt1o and Dnmt1s forms of Dnmt1 protein and their expression in oocytes**. A. Schematic representation of the mouse Dnmt1s and Dnmt1o proteins. Near the N-termini two regions are highlighted, the PCNA binding domain (PBD) and targeting sequence (TS) that have been implicated in targeting Dnmt1 to replication foci [18,29]. The black rectangles identify the positions of the mouse Dnmt1s amino acid sequences used to immunize a rabbit to produce the UPT82 antiserum or to immunize a chicken to produce the UPTC21 antiserum [6]. B. Immunoblots showing expression of Dnmt1s protein in oocytes from various mouse lines. The upper and lower blots were probed with the UPT82 antiserum. + refers to wild-type oocytes; *1s/1o *refers to homozygous mutant oocytes from the *Dnmt1*^1*s*/1*o *^strain [6]; *Δ1o *refers to homozygous mutant oocytes from the *Dnmt1*^Δ1*o *^strain; *TgEL/+ *refers to oocytes from hemizygous *TgEL *transgenic mice. Sample sizes for the upper blot: 100 wild-type GV oocytes; 120 wild-type MII oocytes; 25 *1s/1o *oocytes; 60 *Δ1o *oocytes. Sample sizes for the lower blot: 105 wild-type MII oocytes; 40 *TgEL/+ *oocytes. C. Expression of Dnmt1o protein in wild-type, *1s/1o *and *Δ1o *oocytes. The same blot shown in panel B was probed with UPTC21.

We explored the possibility that the *M*_r _190,000 Dnmt1s form of Dnmt1 is expressed during preimplantation development in wild-type embryos by using an antiserum that binds specifically to Dnmt1s [6], by using appropriate controls for the specificity of immunodetection and by using a different Dnmt1s immunodetection technique. Two different polyclonal antisera were used in these studies. UPT82 is a rabbit polyclonal antiserum that binds to epitopes in the N terminus of Dnmt1s that are not found in the smaller Dnmt1o protein [6]. In contrast, UPTC21 is a chicken polyclonal antiserum that recognizes both the Dnmt1s and the Dnmt1o proteins [6] (Figure 1A).

To examine Dnmt1s expression during oogenesis, immunoblots were produced using extracts from different types of oocytes, and these were first probed with UPT82. To ensure detection of Dnmt1s protein and the accurate comparison of Dnmt1s in different developmental stages, we used extracts of approximately 100 wild-type oocytes per lane. Dnmt1s was present at an approximately 10-fold higher concentration in GV-stage oocytes compared to MII oocytes (Figure 1B). Significant increases in the level of Dnmt1s were seen in homozygous MII oocytes of two different mutant mouse lines, the *Dnmt1*^1*s*/1*o *^line that synthesizes both the Dnmt1s and Dnmt1o proteins from a single, modified *Dnmt1o *transcript [6], and the *Dnmt1*^Δ1*o *^line that does not produce the Dnmt1o protein [5]. The approximately 40-fold increase in Dnmt1s concentration in Dnmt1o-deficient oocytes compared to wild-type MII oocytes most likely is due to an increase in Dnmt1s synthesis. In addition, more Dnmt1s protein is present in MII oocytes from the *TgEL *transgenic line that expresses Dnmt1s from transcripts synthesized using the transgene's *zona pellucida 3 *(*Zp3*) promoter (Figure 1B). A high level of Dnmt1o protein expression in wild-type GV-stage and MII oocytes, a relatively lower level of Dnmt1o in homozygous *Dnmt1*^1*s*/1*o *^MII oocytes, and the absence of Dnmt1o expression in homozygous *Dnmt1*^Δ1*o*/Δ1*o *^MII oocytes were shown using the UPTC21 antiserum (Figure 1C). The absence of detectable oocyte Dnmt1s protein with UPTC21 indicates that UPT82 is more sensitive than UPTC21 in detecting the Dnmt1s protein.

### Dnmt1s is present throughout preimplantation development

**Figure 2:**
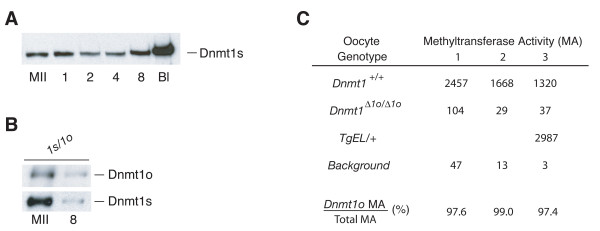
**Dnmt1s protein expression during preimplantation development**. A. Relative amount of Dnmt1s protein in fully grown wild-type MII oocytes, and in embryos at different stages of development. MII = MII oocytes; 1 = 1-cell; 2 = 2-cell; 4 = 4-cell; 8 = 8-cell; Bl = blastocyst. Sample sizes: 105 wild-type MII oocytes; 105 1-cell embryos; 110 2-cell embryos; 110 4-cell embryos; 110 8-cell embryos; 120 blastocysts. B. Relative stability of oocyte-derived Dnmt1o and Dnmt1s proteins from homozygous *Dnmt1*^1*s*/1*o *^mice during preimplantation development to the 8-cell stage. Sample sizes: 22 MII oocytes; 21 8-cell embryos. The blot was first probed with UPT82, stripped and then reprobed with UPTC21. The assay was repeated, and nearly identical results were obtained (data not shown). C. Relative levels of maintenance methyltransferase activity (MA) per oocyte. MA is expressed as [^3^H]methyl group incorporation into the synthetic template poly(dIdC). Oocyte genotype is the genotype of the diploid precursor of the analyzed MII oocyte. The assay was performed on three separate occasions (1–3). The background measurement is the cpm in sample containing poly(dIdC) and S-adenosyl methionine, but no oocyte extract. Dnmt1o MA/Total MA was calculated as [(*Dnmt1*^+/+ ^– background) – (*Dnmt1*^Δ1*o*/Δ1*o *^– background)]/(*Dnmt1*^+/+ ^– background), expressed as a percentage.

Dnmt1s protein was also observed in all preimplantation embryo stages examined (Figure 2A). These included 1-cell, 2-cell, 4-cell, 8-cell embryos, as well as blastocysts. The concentration of Dnmt1s in 1-cell embryos was similar to that seen in wild-type MII oocytes. A nadir of Dnmt1s concentration was seen in wild-type 2-cell and 4-cell embryos. Thereafter, the preimplantation Dnmt1s concentration rose to a maximum in blastocysts. The concentration of Dnmt1s in the blastocyst was approximately 7 times the MII-oocyte concentration and approximately 18 times the 4-cell concentration. The pattern of changes in Dnmt1s protein concentration seen throughout preimplantation development suggests that preimplantation Dnmt1s is derived from a combination of maternal and zygotic protein synthesis. The source of an embryo's Dnmt1s would most likely change from maternal to zygotic near the nadir of Dnmt1s concentration, namely at the 2-cell or 4-cell cleavage stages. This notion is consistent with previous observations in which *Dnmt1s *mRNA is present in MII oocytes and throughout preimplantation development, with a nadir in *Dnmt1s *mRNA concentration at the 1-cell stage [6].

The Dnmt1o and Dnmt1s proteins present in the MII oocyte may serve different functions in early embryos. Dnmt1o is a maternal-effect protein that maintains genomic imprints during preimplantation development, probably at the 8-cell stage where Dnmt1o traffics into nuclei [5]. Maternal Dnmt1s might provide a different function in maintaining DNA methylation in the early embryo, perhaps earlier than the 8-cell stage. In support of this notion, the concentrations of Dnmt1o and Dnmt1s change in very different ways from the MII oocyte stage to the 8-cell stage. 8-cell Dnmt1o is one-sixth the level of Dnmt1o seen in MII oocytes, whereas the concentration of Dnmt1s in 8-cell embryos is only 1/30th that of MII-oocyte Dnmt1s. This represents a 5-fold higher rate of decline in Dnmt1s protein concentration than Dnmt1o's rate of decline (Figure 2B). 8-cell Dnmt1o protein is likely to be entirely oocyte-derived, because Dnmt1o transcripts disappear from the embryo after the 1-cell stage [6]. The relatively slow rate of decline in Dnm1o protein concentration during the first half of preimplantation development is consistent with a highly stable protein, similar to the highly stable nature of Dnmt1o when it is expressed in somatic tissues of *Dnmt1*^*V *^mice [8]. In contrast, the Dnmt1s protein appears to be much less stable during preimplantation development. Oocyte-derived Dnmt1s may be even less stable than indicated by the oocyte/8-cell ratio; any Dnmt1s synthesized in the early preimplantation embryo would contribute to the 8-cell Dnmt1s level, and falsely raise the calculated stability of MII-oocyte Dnmt1s protein. We conclude from this analysis that MII-oocyte Dnmt1s is unstable during the first half of preimplantation development (by at least a factor of five) compared to MII-oocyte Dnmt1o protein.

Dnmt1o protein in MII oocytes was readily detected on immunoblots using the UPTC21 antibody, but oocyte Dnmt1s was not detected using UPTC21 (Figure 1C). Because of the inability to detect both forms of the protein in oocytes with the same antibody, a quantitative difference in the concentrations of the two proteins was not established. To indirectly establish an estimate of a functional difference between the two forms in oocytes, we measured the maintenance methyltransferase activity in oocytes of difference genotypes. As shown in Figure 2C, 97.4% to 99.0% of MII oocyte maintenance methyltransferase activity was due to the presence of the Dnmt1o protein. This represents an approximately 50-fold reduction of maintenance methyltransferase activity in homozygous *Dnmt1*^Δ1*o*/Δ1*o *^oocytes compared to wild-type oocytes. The approximately 2% of normal MII oocyte maintenance activity not attributed to Dnmt1o is probably Dnmt1s activity. Because the oocyte concentration of Dnmt1s was increased by a factor of 40 in the absence of Dnmt1o (Figure 1B), the amount of methyltransferase activity actually attributable to Dnmt1s in wild-type oocytes is more likely to be 0.05% of the total maintenance methyltransferase activity. In cultured somatic cells, the specific maintenance methyltransferase activities of Dnmt1s and Dnmt1o are the same [9]. If we assume that the specific activities are also the same in MII oocytes, then we can infer that the concentration of the Dnmt1s protein in MII oocytes is approximately 1/2000th of the Dnmt1o protein concentration. Such a low relative abundance of Dnmt1s protein probably accounts for our inability to previously detect it in wild-type oocytes [6].

The normal oocyte maintenance methyltransferase activity can be increased approximately 2-fold in *TgEL *oocytes (Figure 2C). *TgEL *MII oocytes had approximately 13 times the level of Dnmt1s protein compared to wild-type MII oocytes when the protein levels are estimated from an immunoblot probed with the UPT82 antibody (Figure 1B). The cause of this discrepancy between the modest increase in maintenance methyltransferase activity and the large increase in protein level relative to the wild-type amount of Dnmt1o protein is unknown. *TgEL*-derived Dnmt1s protein contains an additional 46 amino acids at its amino terminus, and these amino acids are adjacent to the 118-aa region containing epitopes detected by the UPT82 antibody. It is unlikely that the addition of 46 amino acids to the amino terminus of Dnmt1s would influence its innate biochemical methyltransferase activity. It is more likely that either the additional 46 amino acids negatively affect the sensitivity of detection of the transgene protein or that the ooplasmic transgene protein is more enzymatically active than the abundant, stored Dnmt1o protein. The latter effect could occur if the majority of the ooplasmic Dnmt1o is enzymatically inactive, whereas the majority of the transgene Dnmt1s protein is enzymatically active.

### Intracellular localization of preimplantation Dnmt1s protein

**Figure 3:**
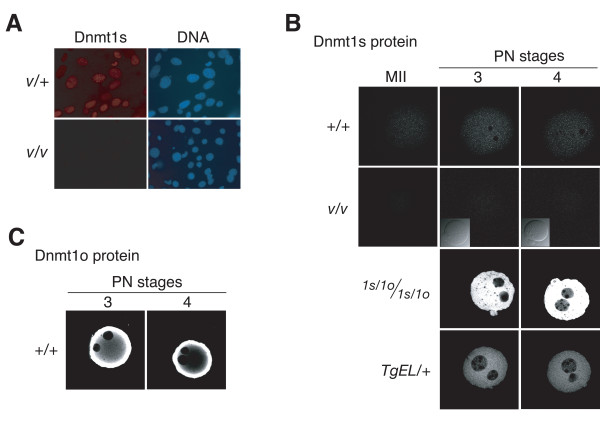
**Immunolocalization of Dnmt1o and Dnmt1s proteins in mouse embryonic fibroblasts and 1-cell embryos**. A. Immunostaining of primary embryonic fibroblasts from either heterozygous *Dnmt1*^*V*/+ ^(*V/+*) or homozygous *Dnmt1*^*V*/*V *^(*V/V*) mice using the UPT82 antiserum. Intracellular DNA was detected with DAPI. B. Immunostaining of MII oocytes and pronuclear-stage embryos of different genotypes with the UPT82 antiserum. PN3 and PN4 refer to pronuclear stage 3 and 4 as defined by morphologic criteria [17]. Insets are bright-field images of immunostained embryos. C. Immunostaining of pronuclear-stage embryos with the UPTC21 antiserum to detect the abundant Dnmt1o protein. Genotype abbreviations are the same as in the legends to Figures 1 and 2. Bar, 20 μm.

**Figure 4:**
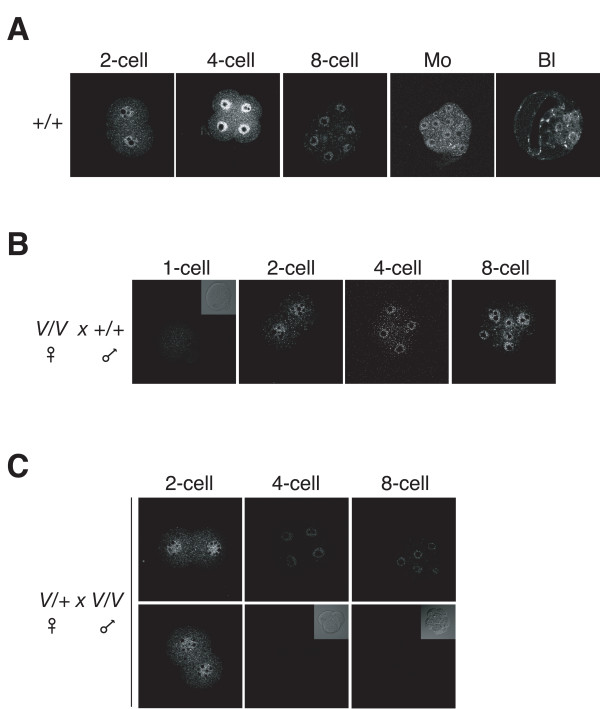
**Expression of maternal and zygotic Dnmt1s during preimplantation development**. A. Time course of Dnmt1s expression at defined preimplantation cleavage stages determined by immunostaining with the UPT82 antiserum. Mo = morula. Bl = blastocyst. B. Time course of Dnmt1s expression during preimplantation development in embryos derived from crosses between wild-type (+/+) female mice and homozygous *Dnmt1*^*V*/*V *^(V/V) male mice. Inset is bright-field image of immunostained 1-cell embryo. C. Time courses of Dnmt1s expression during preimplantation development in embryos derived from crosses between heterozygous *Dnmt1*^*V*/+ ^(V/+) female mice and homozygous *Dnmt1*^*V*/*V *^(V/V) male mice. The 4-cell and 8-cell embryos in the top row show primarily nuclear Dnmt1s staining whereas 4-cell and 8-cell embryos in the botton row show little or no nuclear Dnmt1s. All seven 2-cell, three out of seven 4-cell and six out of 13 8-cell embryos showed nuclear staining. Bar, 20 μm.

As shown in Figure 3A, the UPT82 antiserum stains nuclei of mouse embryonic fibroblasts (MEFs) from heterozygous *Dnmt1*^*V*/+ ^embryos, but does not stain nuclei of homozygous *Dnmt1*^*V*/*V *^MEFs. Moreover, the Dnmt1s protein is not detected on immunoblots of homozygous *Dnmt1*^*V*/*V *^somatic tissues probed with the UPTC21 antibody [8]. These observations indicate that UPT82 specifically identifies Dnmt1s in cells. In light of these observations, we stained wild-type pronuclear-stage 1-cell embryos with UPT82 and showed that Dnmt1s is present throughout the cytoplasm. Nuclear staining was not observed. As expected, no staining at all was evident in homozygous *Dnmt1*^*V*/*V *^embryos derived from crosses between a *Dnmt1*^*V*/*V *^female and a *Dnmt1*^*V*/*V*^male. The specific cytoplasmic staining seen in 1-cell embryos was true of early pronuclear stages (data not shown for PN1 and PN2 stages), as well as the later PN3 and PN4 stages (Figure 3B). In PN-stage 1-cell embryos derived from *Dnmt1*^1*s*/1*o *^and *TgEL *oocytes, which produce greater than normal amounts of Dnmt1s, cytoplasmic Dnmt1s staining was significantly higher than nuclear Dnmt1s staining (Figure 3B). Finally, when pronuclear-stage 1-cell embryos were stained with the UPTC21 antibody, there was intense cytoplasmic staining for Dnmt1o, but no pronuclear staining was evident (Figure 3C). This cytoplasmic localization of Dnmt1o has been previously described [10,11]. Dnmt1s staining was seen in wild-type 2-cell, 4-cell and 8-cell embryos, as well as in nuclei of wild-type morulae and blastocysts (Figure 4A). In contrast to the cytoplasm-specific staining of pronuclear-stage 1-cell embryos, the staining of the later stages was concentrated in the nuclei. We conclude from this analysis that Dnmt1s protein is expressed in all stages of preimplantation development, primarily in nuclei of cells at all stages except the pronuclear stages of the 1-cell embryo.

### Dnmt1s is derived from two sources

One likely cause for the varying levels of Dnmt1s protein during preimplantation development (Figure 2A) would be that levels during this developmental window are determined by a combination of oocyte (maternal) synthesis and post-fertilization (zygotic) synthesis. To explore this possibility, preimplantation embryos derived from crosses between wild-type and *Dnmt1*^*V *^mice were examined. In 1-cell, 2-cell, 4-cell and 8-cell embryos derived from crosses between homozygous *Dnmt1*^*V*/*V *^mutant female mice and wild-type male mice, Dnmt1s was present in 2-cell, 4-cell and 8-cell embryos (including in the nuclei), but not in 1-cell embryos (Figure 4B). Because these embryos should only synthesize Dnmt1s zygotically from the paternal allele, we interpret these results as indicating that the Dnmt1s in wild-type 1-cell embryos is of maternal (oocyte) origin, and Dnmt1s protein seen afterwards at least partially originates from zygotic (post-fertilization) synthesis.

To establish the duration of expression of the maternally derived Dnmt1s protein beyond the 1-cell stage, we examined embryos from crosses between heterozygous *Dnmt1*^*V*/+ ^females and homozygous *Dnmt1*^*V*/*V *^males. One-half of embryos from this cross will synthesize only maternal Dnmt1s, whereas the other half will synthesize Dnmt1s both maternally and zygotically. When a series of 2-cell embryos were examined, all stained for cytoplasmic and nuclear Dnmt1s (Figure 4C). However, when a series of 4-cell and a series of 8-cell embryos were stained, approximately one-half of the embryos expressed both nuclear and cytoplasmic Dnmt1s, whereas the other half showed no significant staining (Figure 4C). These results are consistent with expression of maternal Dnmt1s through the 2-cell stage, but not beyond. Taken together with the results of Dnmt1s expression in offspring of crosses between wild-type females and *Dnmt1*^*V*/*V *^males, these findings are also consistent with a zygotic origin of Dnmt1s found in 2-cell embryos and beyond.

### Oocyte-derived Dnmt1s restores imprinting in Dnmt1o-deficient embryos

Both the Dnmt1s and the Dnmt1o forms of Dnmt1 protein are present in nuclei of 8-cell blastomeres (Figures 4A) [7]. Despite this, in embryos derived from homozygous mutant *Dnmt1*^Δ1*o*/Δ1*o *^female mice, loss of Dnmt1o from nuclei of 8-cell blastomeres was not functionally compensated for Dnmt1s. It would appear therefore that Dnmt1s in nuclei of 8-cell blastomeres does not function to maintain methylation patterns on differentially methylated domains (DMDs) associated with imprinted genes. Because 8-cell nuclear Dnmt1o is of maternal origin and 8-cell nuclear Dnmt1s is of zygotic origin, it is possible that the zygotic origin of 8-cell nuclear Dnmt1s precludes its function in nuclei of 8-cell embryos. In view of this, we explored the possibility that oocyte-derived Dnmt1s can replace Dnmt1o in maintaining genomic imprints.

**Figure 5:**
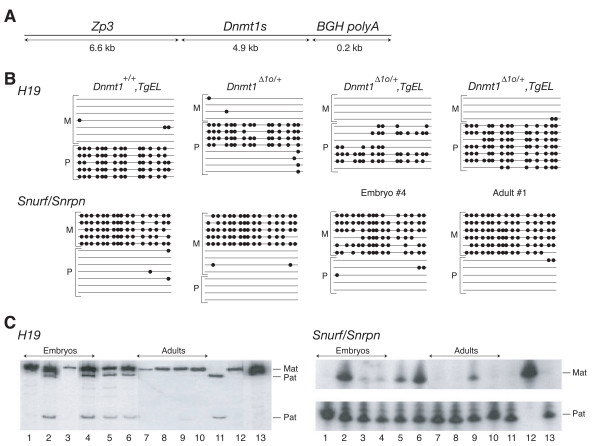
**The forced expression of maternal Dnmt1s protein prevents loss of imprinting due to the absence of maternal Dnmt1o protein**. A. Schematic of construct used to force expression of an epitope-tagged version of Dnmt1s in fully grown mouse oocytes of the *TgEL *transgenic mouse line. ZP3 = 6.6-kb mouse zona pellucida 3 gene promoter [23]. Dnmt1s = full-length Dnmt1s cDNA containing coding sequence for N-terminal Anti-Xpress epitope tag. BGH = bovine growth hormone polyadenylation and transcription termination sequence. B. Summary of bisulfite genomic sequencing of two normally imprinted genes in mice of various genotypes. *Dnmt1*^Δ1*o*/+ ^alleles were analyzed in a DNA sample isolated from an E13.5 embryo derived from a cross between a homozygous *Dnmt1*^Δ1*o*/Δ1*o *^female and a Cast-7 male. *Dnmt1*^Δ1*o*/+^, *TgEL *alleles were analyzed in DNA samples isolated either from an E13.5 embryo (Embryo #4) or from an adult (Adult #1) derived from crosses between a *Dnmt1*^Δ1*o*/Δ1*o*^, *TgEL *female and a Cast-7 male. C. Analysis of *H19 *and *Snurf/Snrpn *gene expression in mice of various genotypes. Allele-specific expression of *H19 *was determined by restriction endonuclease digestion of RT-PCR products. Allele-specific expression of *Snurf/Snrpn *was determined by SnuPE assay. Lanes 1–4: *Dnmt1*^Δ1*o*/+^, *TgEL *E13.5 embryos, Embryo #1 through Embryo #4. Lane 5–6: *Dnmt1*^Δ1*o*/+ ^embryos. Lanes 7–10: *Dnmt1*^Δ1*o*/+^, *TgEL *adults, Adult #1 through Adult #4. Lane 11: inbred Cast-7. Lane 12: inbred FVB/N. Lane 13: F1 hybrid from cross between an FVB/N female mouse and a Cast-7 male mouse.

The *TgEL *transgenic line expresses an epitope-tagged version of Dnmt1s in MII oocytes (Figures 1B and 5A). The *TgEL*-derived Dnmt1s is likely to be catalytically active *in vivo*, because *TgEL *MII oocytes have increased maintenance methyltransferase activity (Figure 2C). The ability of the *TgEL *oocyte-derived Dnmt1s to maintain embryonic imprints in the absence of Dnmt1o was tested by crossing *Dnmt1*^Δ1*o*/Δ1*o*^, *TgEL/+ *mice with Cast-7 mice. The *Dnmt1*^Δ1*o *^allele and the *TgEL *transgene were both crossed into the inbred FVB/N strain background. Nine crosses each between *Dnmt1*^Δ1*o*/Δ1*o *^females or *Dnmt1*^Δ1*o*/Δ1*o*^, *TgEL *females and Cast-7 males were established and maintained for 9 months. Six litters of live newborns were obtained from the *Dnmt1*^Δ1*o*/Δ1*o*^, *TgEL *females, whereas no live newborns were obtained from the *Dnmt1*^Δ1*o*/Δ1*o *^females. Only a total of 14 live offspring were obtained from the six litters born to *Dnmt1*^Δ1*o*/Δ1*o*^, *TgEL *female mice (litters of 4, 4, 2, 2, 1, and 1 offspring). These results indicate that the presence of the *TgEL *transgene in the female parent had a beneficial effect on recovering viable offspring.

To explore the possibility that the beneficial effect of Dnmt1s expression in Dnmt1o-deficient oocytes was due to a Dnmt1s rescue of Dnmt1o-catalyzed maintenance of genomic imprints in the early embryo, we examined the methylation and expression of imprinted genes in offspring of *Dnmt1*^Δ1*o*/Δ1*o*^, *TgEL *female mice. Methylation of *H19 *and *Snurf/Snrpn *DMDs was measured in four E13.5 embryos and four surviving adults (from a single litter) derived from crosses between *Dnmt1*^Δ1*o*/Δ1*o*^, *TgEL *females and wild-type Cast-7 males. As shown in Figure 5B, when one E13.5 embryo (Embryo #4) and one adult (Adult #1) were examined in detail, more paternal *H19 *alleles and more maternal *Snurf/Snrpn *alleles were methylated in the adult offspring than in the embryo. Stated differently, the level of DNA methylation of Embryo #4 is more similar to the level of methylation of an E13.5 embryo derived from a *Dnmt1*^Δ1*o*/Δ1*o *^female mouse (Figure 5B) [5], whereas the level of methylation of the *H19 *gene of Adult #1 is more similar to the level of methylation in an adult mouse derived from a *TgEL *female mouse (Figure 5B). This type of analysis was carried out on an additional three embryos and three adults, and the data summarized in Table 1. In most instances the level of methylation among both embryos and adults from *Dnmt1*^Δ1*o*/Δ1*o*^, *TgEL *female mice was not significantly different than the extent of *H19 *paternal allele and *Snurf/Snrpn *maternal allele methylation in a wild-type offspring from a cross between an FVB/N female and a Cast-7 male. We conclude from this analysis that a significant rescue of the methylation defect due to the absence of the oocyte's Dnmt1o protein occurred when Dnmt1s protein was overexpresed in fully grown oocytes.

**Table 1:** 

		Percentage Methylated CpGs			
**Mouse Genotype**^a^	**Embryo/Adult Number**	***H*19**		***Snurf/Snrpn***	
		
		**Mat**	**Pat**	**Mat**	**Pat**
*Dnmt*1^+/+^		3	93	91	0
*Dnmt*1^Δ1*o*/+^		3	57^d^	44^d^	1
*TgEL*/+		2	92^b^	92^b^	4
	Embryo 1	9	88^b^	89^c^	4
	Embryo 2	13	69^c^	79^b^	4
	Embryo 3	8	87^b^	73^b^	1
*Dnmt*1^Δ1*o*/+^, *TgEL*/+	Embryo 4	0	54^c^	84^b^	2
	Adult 1	0	71^b^	93^b^	3
	Adult 2	1	87^b^	77^b^	11
	Adult 3	17	85^b^	93^b^	5
	Adult 4	11	82^b^	96^b^	0

^a ^*Dnmt*1^Δ1*o*/+ ^embryo is from a cross between a *Dnmt*1^Δ1*o*/Δ1*o *^female mouse and a Cast-7 male; *Dnmt*1^Δ1*o*/+^, *TgEL *embryo is from a cross between a *Dnmt*1^Δ1*o*/Δ1*o*^, *TgEL *female mouse and a Cast-7 male.

^b ^No significant difference compared to *Dnmt*1^+/+ ^methylation

^c ^Significant difference compared to *Dnmt*1^+/+ ^methylation (p < 0.05)

^d ^Significant difference compared to *Dnmt*1^+/+ ^methylation p < 0.025)

The improvement in *H19 *paternal allele methylation and *Snurf/Snrpn *maternal allele methylation in offspring when oocyte Dnmt1o is replaced with Dnmt1s is associated with an improvement in imprinted gene expression. In the same four embryos derived from *Dnmt1*^Δ1*o*/Δ1*o*^, *TgEL *females, in which allele-specific DNA methylation was examined, two of the four embryos (Embryo #2 and Embryo #4) showed abnormal biallelic expression of *H19*. The remaining two embryos (Embryos #1 and Embryo #3) showed the normal monoallelic *H19 *expression from the maternal allele (Figure 5C). One of the embryos (Embryo #2) with biallelic *H19 *expression also showed biallelic *Snurf/Snrpn *expression. The other three embryos showed monoallelic expression or near monoallelic expression from the paternal *Snurf/Snrpn *allele. In contrast, all four of the examined adult mice from *Dnmt1*^Δ1*o*/Δ1*o*^, *TgEL *female mice showed normal monoallelic *H19 *expression. Only one of these (Adult #3) showed evidence of abnormal expression of *Snurf/Snrpn *from both parental alleles. We conclude from this analysis that oocyte-derived Dnmt1s is capable of a complete or partial functional substitution of the normal oocyte Dnmt1o protein.

## Discussion and Conclusions

### Dnmt1 and the inheritance of genomic imprints

Imprinted genes have stringent epigenetic requirements. Arguably, the most important of these is the inheritance of highly methylated alleles whose parent-specific methylation was established during gametogenesis [1]. The inheritance of this gametic methylation is particularly noteworthy during preimplantation development. Here, highly methylated alleles maintain their methylation in the face of significant rearrangements of genomic DNA methylation. These rearrangements include the loss of methylation on non-imprinted gene sequences, the maintenance of CpG methylation on classes of repetitive sequences, and a significant overall loss of gametic methylation [12-14].

The DNA sequences carrying the inherited imprinted methylation are called differentially methylated domains (DMDs) [1]. DMD methylation must be accurately inherited at every S phase of every embryonic cell cycle in order to sustain the DMD methylation in every cell of the developing embryo. This may be particularly true of preimplantation development. Here, the consequences of not inheriting DMD methylation for even one S phase are severe, as exemplified by a 50% reduction of imprinted methylation and fetal death in the absence of the maternal-effect Dnmt1o protein [5]. One can easily imagine that the loss of other components of the machinery controlling the inheritance of DMD methylation in the preimplantation embryo would lead to similar catastrophic effects.

Most of the molecular machinery that ensures the inheritance of imprinted DMD methylation during preimplantation development is unknown. At the very least, maintenance methyltransferase activity would be required at every S phase. There are approximately seven S phases of preimplantation development, and the maintenance methyltransferase activity of only one of these S phases has been identified. This is the fourth S phase, whose methyltransferase activity is attributed to the oocyte-derived Dnmt1o protein [5]. We had previously only been able to observe Dnmt1s in 4-cell, 8-cell and 16-cell abnormal embryos in which Dnmt1o protein was absent [7]. We have now shown here that Dnmt1s is present throughout preimplantation development, and present in the nucleus at nearly all of these stages. In support of this, the presence of preimplantation Dnmt1s protein has been recently reported by other investigators [15]. Because of this, we propose that Dnmt1s maintains imprinted DNA methylation patterns at the other S phases of preimplanation development.

### Different forms of Dnmt1

**Figure 6:**
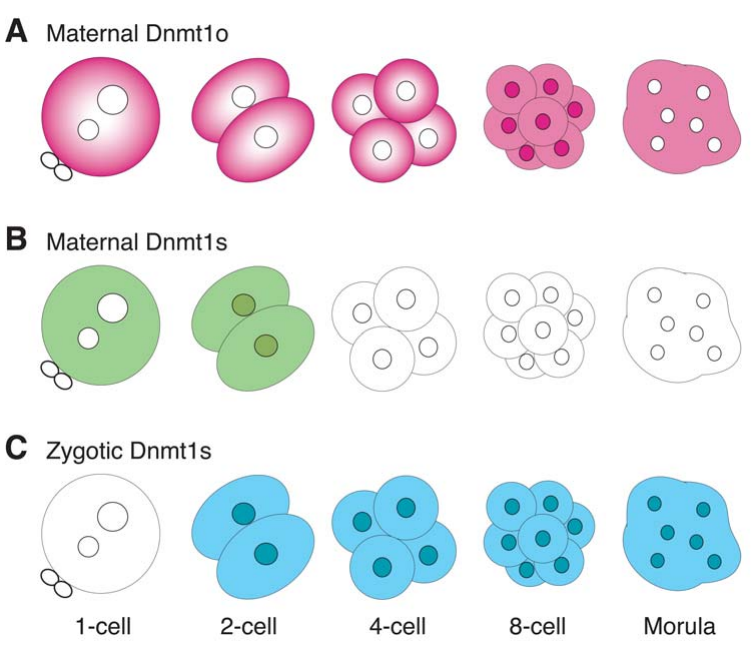
**Model of Dnmt1o and Dnmt1s protein expression during preimplantation development**. A. The top row of schematics depicts the pattern of maternal Dnmt1o protein expression seen during preimplantation development. Dnmt1o is localized in the nucleus of blastomeres of 8-cell embryos. Dnmt1o is not found in the pronuclei. B. The middle row of schematics depicts the pattern of maternal Dnmt1s protein expression in the early stages of preimplantation development. Dnmt1s is not found in the pronuclei. C. The bottom row of schematics depicts the pattern of zygotic Dnmt1s protein expression during preimplantation development. Dnmt1s is evident in nuclei of many stages, including in blastomeres of 8-cell embryos.

Dnmt1 proteins found in oocytes and preimplantation embryos are primarily distinguished by size. In the mouse, the *M*_r _190,000 Dnmt1s protein contains the entire peptide sequence of the *M*_r_175,000 Dnmt1o protein; the larger size of Dnmt1s is due to an additional 118 amino acids at the amino terminus (Figure 1A). In the wild-type mouse, Dnmt1o is synthesized only in the oocyte, and functions in the inheritance of genomic imprints when it traffics into the nuclei of 8-cell embryos (fourth S phase). As shown here, Dnmt1s is synthesized in both oocytes and preimplantation embryos. These observations suggest that the combination of Dnmt1o and Dnmt1s proteins from the oocyte, and Dnmt1s protein from the preimplantation embryo work together to ensure the accurate inheritance of DMD methylation (Figure 6).

As indicated above, in addition to Dnmt1 proteins of different sizes, Dnmt1 proteins also come from different developmental sources. This raises the issue as to whether oocyte and preimplantation Dnmt1 proteins are primarily distinguished in their functions by amino acid sequence or by their developmental source (oocyte versus embryo). Two experiments indicate that the important distinction between the Dnmt1o and Dnmt1s proteins is most likely their different developmental sources. First, an oocyte-derived, epitope-tagged version of Dnmt1s restores imprinting in the absence of Dnmt1o (Figure 5 and Table 1). This indicates that oocyte-derived Dnmt1s functions in the absence of oocyte-derived Dnmt1o. Presumably, when Dnmt1s is synthesized to levels significantly greater than found in wild-type oocytes, it can persist in sufficient quantities to the 8-cell stage, traffick into the blastomere nuclei and maintain genomic imprints. The inefficiency of this maintenance is likely due to the difference in preimplantation stabilities between oocyte Dnmt1o and Dnmt1s (Figure 2B). A marked difference in stability of the two proteins is also seen in heterozygous *Dnmt1*^*V *^somatic cells that synthesize both Dnmt1s and Dnmt1o proteins [8].

The second observation supporting a functional difference between maternally and zygotically synthesized Dnmt1 proteins is the viability of homozygous *Dnmt1*^*V*/*V *^mice. Dnmt1o protein expressed from the *Dnmt1*^*V *^alleles in these homozygous *Dnmt1*^*V*/*V *^mice maintains inherited methylation patterns in the absence of both maternal and zygotic Dnmt1s. The interchangeability of Dnmt1o and Dnmt1s proteins during preimplantation development is inconsistent with a meaningful difference in maintenance methyltransferase function of the two proteins. It is consistent, however, with a primary functional difference between maternal (oocyte-synthesized) and zygotic (embryo-synthesized) Dnmt1 proteins.

### Potential functions of Dnmt1 proteins

The 8-cell preimplantation embryo highlights the functional difference between oocyte- and embryo-derived Dnmt1 proteins. The fourth cycle of embryonic DNA replication occurs at this cleavage stage. In all likelihood, methylation of DMD sequences is maintained immediately following DNA replication at this fourth S phase. Although both the Dnmt1o and the Dnmt1s proteins are present in nuclei of 8-cell blastomeres (Figure 4) [5], only the Dnmt1o protein provides maintenance methyltransferase activity to maintain DMD methylation. A significant loss of DMD methylation occurs even though Dnmt1s is seen in nuclei of 8-cell *Dnmt1*^Δ1*o *^mutant embryos that are devoid of Dnmt1o [7,5]. Because of this, we can conclude that the persistent zygotically synthesized Dnmt1s protein is unable to replace the normal Dnmt1o maintenance function.

An unexpected observation of these studies was the absence of Dnmt1s and Dnmt1o proteins in the pronuclei of 1-cell embryos (Figures 3B and 3C). Dnmt1s is seen only in the cytoplasm of all pronuclear-stage embryos, including mutant ones that express more Dnmt1s protein, and the abundant Dnmt1o protein is only in the cytoplasm of wild-type pronuclear-stage embryos. Both proteins must, therefore, not be imported into the pronuclei or be actively exported from the pronuclei into the cytoplasm of the 1-cell embryo.

DNA replication occurs during the latter stages (PN3 and PN4) of pronuclear changes, prior to the breakdown of the pronuclear membranes [16,17]. This indicates that, if Dnmt1s provides the maintenance methyltransferase function in association with the first replication cycle, it does so after pronuclear breakdown. Dnmt1 maintenance methyltransferase activity is generally closely associated with DNA replication, both in its timing and the intranuclear co-localization of Dnmt1 with replication machinery [18]. Therefore, a delay between DNA replication and maintenance methylation in 1-cell embryos would be unexpected [19]. Moreover, given the brevity of the G2 phase of the first embryonic cell cycle [13], the absence of pronuclear Dnmt1 proteins might indicate that maintenance methylation actually occurs in the G1 phase of the next (second) embryonic cell cycle.

Except for the nuclei of blastomeres of 8-cell embryos, embryo-derived Dnmt1s is the sole nuclear Dnmt1 protein from the 4-cell embryo stage, onward. Therefore, it would appear to be an ideal candidate for catalyzing the maintenance of DMD methylation at the corresponding S phases. However, if embryo-derived Dnmt1s cannot maintain imprinted DMD methylation at the fourth S phase, can it maintain imprinted DMD methylation at other S phases? The answer to this question is unknown. However, if it does indeed maintain DMD methylation at stages other than the 8-cell stage, this suggests that the same protein functions differently in the nucleus at different cleavage stages, or alternatively that the Dnmt1s protein in the nucleus at a non-8-cell stage is not the same as the Dnmt1s protein at the 8-cell stage. Very different models are needed to explain these two possibilities. In the first case, factors other than the Dnmt1s protein itself, such as DNA structural differences or proteins other than Dnmt1, would have to be involved as part of the maintenance mechanism. In the second case, possibly the more interesting one, processes than expand the actual variety of Dnmt1s proteins, would have to be invoked. Processes to consider are transcriptional or post-transcriptional processes that provide a variety of mature Dnmt1s transcripts or post-translational processes that directly modify Dnmt1s proteins. In this regard, splicing variants of Dnmt1 transcripts that produce variant Dnmt1 proteins, and post-translational serine phosphorylation of Dnmt1s have been reported to occur in adult somatic cells [20-22,19]. Whether similar processes that can expand the forms of Dnmt1 proteins exist in oocytes or preimplantation embryos is presently unknown.

Taken together our results indicate that Dnmt1s is expressed in all diploid-nuclear stages of mouse preimplantation embryos, suggesting that Dnmt1s provides maintenance methylation activity for the inheritance of methylation imprints in the early mouse embryo. The combination of Dnmt1o and Dnmt1s proteins from the oocyte and Dnmt1s protein from the preimplantation embryo work together to ensure the accurate inheritance of genomic imprints.

## Methods

### Animals

The mutant *Dnmt1*^1*s*/1*o *^allele was constructed by inserting coding sequences for the amino terminus of the Dnmt1s protein into exon 1o of the *Dnmt1 *gene [6]. The *Dnmt1*^Δ1*o *^allele was generated by the targeted deletion of exon 1o [5]. The *Dnmt1*^*V *^allele was constructed by mutating the Dnmt1s initiation codon in exon 1s of the Dnmt1s gene [8]. *Dnmt1*^1*s*/1*o *^and *Dnmt1*^*V *^alleles were maintained in the C57Bl/6 background, and the *Dnmt1*^Δ1*o *^allele was maintained in the inbred FVB/N background. Cast-7, an inbred consomic C57Bl/6 strain containing a *M. mus castaneus *chromosome 7, was provided by Dr. Marisa Bartolomei. All experiments on mice conformed to the policies of the University of Pittsburgh Institutional Animal Care and Use Committee.

The DNA fragment to produce the *TgEL *transgenic mouse line was constructed using a modified pcDNA3.1/HisB plasmid (Invitrogen). The CMV promoter of pcDNA3.1 was replaced with a 6.5-kb mouse *Zp3 *promoter [23], and a full-length mouse *Dnmt1 *cDNA was cloned into the unique *Eco*RV site, adjacent to sequences coding for the Anti-Xpress Epitope tag. A DNA fragment (Zp3-Dnmt1-BGH) was released from the plasmid and injected into pronuclei of inbred FVB/N zygotes. The *TgEL *line was maintained in an inbred FVB/N background, except when *TgEL*-containing lines were crossed to Cast-7 male mice.

Fully grown, germinal vesicle (GV)-stage oocytes were obtained from ovarian follicles of 21- to 35-day-old females. Metaphase II (MII) oocytes were collected from females superovulated by injection of pregnant mare serum gonadotropin (PMSG) (Calbiochem, LaJolla, CA), followed 44–48 hours later by human chorionic gonadotropin (hCG) (Sigma) and cumulus cells were dispersed with 1 mg/ml hyaluronidase (Sigma). Preimplantation embryos at the 2-cell stage were isolated from the oviducts at 1.5 days post coitum (dpc); 4-cell embryos; 8-cell embryos and morulae were isolated from oviducts between 2 and 3 dpc. Blastocysts were isolated from the uterus at 3.5 dpc. All oocytes and embryos were collected in M2 medium (Specialty Media) followed by a wash in 1× PBS.

### Immunoblotting

Oocytes and preimplantation embryos were lysed in 0.1 M Tris HCl (pH 6.8), 20% glycerol, 2.5% β-mercapthoethanol, 4% SDS, 0.02% bromophenol blue (2× sample buffer). Samples were denatured by heating at 95°C and then separated by electrophoresis on SDS-5% polyacrylamide gels. Afterwards, they were transferred to PVDF membranes (Immobilon-P Millipore). Membranes were blocked in 5% dry skim milk in 0.1% Tween-20 PBS (PBS-T) for 1 hour and probed with UPT82 (1:1,000), or UPTC21 (1:1,000) overnight at 4°C. Following 5 washes of 5 minutes each in PBS-T, the membranes were incubated for 1 hour in donkey anti-rabbit IgG (Amersham) diluted 1:10,000 in blocking solution, or rabbit anti-chicken IgY (Promega) diluted 1:5,000 in blocking solution. Membranes were washed as above. Bound antibody was detected using the chemiluminescence detection kit ECL Plus (Amersham). When a blot was reprobed with a different primary antibody, the first antibody was removed by incubation with stripping solution and then the blot blocked for 1 hour at room temperature. Comparisons in the intensity of antibody binding between any two lanes on the same immunoblot were determined using a PDSI Scanner (Amersham Bioscience) and ImageQuant 5.2 software.

### Immunocytochemistry

Oocytes and preimplantation embryos were freed of the zona pellucida using acidified Tyrode's medium, washed with PBS and transferred to a cover slide treated with poly-L-lysine (Sigma-Aldrich), then fixed for 10 minutes at room temperature in 3.7% formaldehyde in PBS. The fixed cells were blocked for 1 hour in blocking buffer (3% BSA, 0.1% Triton-X100) and then incubated in either UPT82 (1:250) or UPTC21 (1:100), diluted in blocking buffer, overnight at 4°C in a humidified chamber. Following 3 washes of 5 min each in blocking buffer, the samples were incubated in 1:100 of donkey anti-rabbit IgG fluorescein (FITC)-conjugated (Jackson Immunoresearch) or in 1:250 of goat anti-chicken IgG Alexa Fluor 488 (Molecular Probes) for 1 hour, and washed as before. The cover slide with the cells was placed on a glass microscope slide with a drop of Vectashield mounting medium (Vector Laboratories, Burlingame, CA) supplemented with 0.4 ug/ml of the DNA-binding dye DAPI. Immunofluorescence was visualized using a Leica SP2 Laser Scanning confocal microscope using 63X lens. All images were recorded under identical laser conditions.

Fibroblasts from heterozygous *Dnmt1*^*V*/+ ^or homozygous *Dnmt1*^*V*/*V *^embryos were grown on glass coverslips. The cells were permeabilized for 20 minutes in 0.5% Triton X-100 in PBS. UPT82 was used at a concentration of 1:100 in PBS. A goat anti-rabbit secondary antibody conjugated to Texas Red (Molecular Probes) was used. All washes were performed with 1× PBS and incubation and wash times were identical to those used for oocyte and embryo staining described above. Immunofluorescence was visualized with a Leica DMI4000 fluorescence microscope.

### DNA methyltransferase activity and allele-specific DNA methylation

The methyltransferase activity assay, with the following modifications, was adapted from Issa et al. (1993). The reaction mixture was extracted with phenol:chloroform, using phase-lock gels (Eppendorf), and the filters washed eight times with 5% cold trichloroacetic acid and thrice with 70% ethanol. The radioactivity on the filter was measured with a liquid scintillation counter. Pools of 20–40 oocytes were used for each reaction.

Bisulfite genomic sequencing was performed as described [24]. PCR primers to the 5' regions of two genes (*H19*, GenBank accession number U19616, nucleotides 1301–1732; and *Snurf/Snrpn*, GenBank accession number AF081460, nucleotides 2151–2562) were synthesized, and parental alleles were distinguished by single-nucleotide polymorphisms in crosses between FVB/N and Cast-7 strains. DNA was prepared from adult skeletal muscle or brain, or from entire E13.5 embryos. Nested PCR reactions were necessary to obtain sufficient product from the treated genomic DNA. PCR products were electrophoresed on a 1% agarose gel and isolated PCR fragments were subcloned into the TOPO-TA pCR2.1 vector (Invitrogen, Carlsbad, CA) for sequencing. Statistical differences in methylation of CpG dinucleotides of imprinted genes were evaluated using a paired Student T-test.

### Allele-specific gene expression

The same embryos and adult survivors used for methylation analysis were used in studies of imprinted gene expression. RNA was extracted from adult skeletal muscle or brain, or from entire E13.5 embryos by using the UltraSpec RNA extraction kit on frozen and pulverized tissues (Biotecx, Houston, TX). cDNA was synthesized using the M-MLV kit (Promega, Madison, WI). All assays were dependent on the presence of distinguishing polymorphisms between the two parental alleles. Either RNA from whole E13.5 embryos or adult skeletal muscle was used for analysis of *H19 *expression. *H19 *was PCR amplified using specific *H19 *primers, *H19 *forward 5' CCT CAA GAT GAA AGA AAT GGT 3' and H19 reverse 5' AAC ACT TTA TGA TGG AAC TGC 3' [25] in the presence of α- [^32^P]dCTP. The PCR product was digested with *CaC8*I and run on a polyacrylamide gel. Bands were visualized using auto-radiography. RT-PCR products from the Cast-7 allele contained a *CaC8*I restriction site, resulting in a 148-bp band, whereas products from the FVB/N allele did not, resulting in the full-length PCR product of 648 bp. For *Snurf/Snrpn*, RNA from either whole embryos or adult brain was used and expression was analyzed by the single nucleotide primer extension (SNuPE) technique as previously described [26-28,6]. After electrophoresis of the samples on a 15% denaturing gel, bands were visualized by autoradiography. The incorporation of dGTP indicated the expression of the FVB/N allele, while incorporation of dATP indicated the expression of the Cast-7 allele

## Authors' contributions

MCC carried out the immunoblots and immunocytochemistry assays and helped to draft the manuscript. SR performed the experiments with the *TgEL *transgenic mice. FD carried out the methyltransferase activity assays. BR performed the 1s vs 1o stability Western blots. CN helped with the immunocytochemistry experiments. JRC conceived of the study and wrote the manuscript. All authors read and approved the final manuscript.
